# Imposter phenomenon; prevalence and associated factors among family medicine residents in Riyadh city, KSA

**DOI:** 10.1016/j.jtumed.2025.08.001

**Published:** 2025-09-07

**Authors:** Abdulrahman Bamhair, Salem Alshahrani, Mohammed Alqahtani, Abdulelah Alotaibi, Omar Alghamdi

**Affiliations:** King Fahad Medical City, Riyadh, KSA

**Keywords:** Family, Imposter syndrome, Medicine, Resident, Residency program

## Abstract

**Objectives:**

Imposter syndrome (IS) is a psychological phenomenon characterized by persistent self-doubt and fear of being exposed as incompetent despite clear evidence of competence. This study was aimed at determining the prevalence of IS, and identifying associated demographic predictors, among family medicine residents in Riyadh, KSA.

**Methods:**

A cross-sectional study was conducted among family medicine residents from multiple training centers in Riyadh using a self-administered questionnaire that included the Clance Impostor Phenomenon Scale (CIPS). Sociodemographic data were also collected.

**Results:**

A total of 268 residents participated (mean age 26.1 ± 1.8 years; 63.4% males). The mean IS score was 50.8 ± 13.0 with a median of 51 (IQR 41–60). Over half (53.7%, n = 144) were in the moderate IS category, 23.1% (n = 62) reported few IS characteristics, 21.3% (n = 57) experienced frequent IS, and 1.9% (n = 5) had intense IS symptoms. Distribution of IS categories differed significantly across training centers (p < 0.001). Marital status was associated with IS severity (p = 0.037), as married residents more often reported fewer IS characteristics (30.3%) compared with single residents (20.8%). Age (p = 0.684), gender (p = 0.714), and first-generation physician status (p = 0.461) showed no significant associations.

**Conclusion:**

IS was highly prevalent among family medicine residents in Riyadh, with most experiencing moderate-to-frequent symptoms. Considering factors such as residency year and marital status, and providing robust psychosocial support, may help reduce IS severity. Future studies should use longitudinal designs to explore causal pathways and extend findings to other medical specialties and regions.

## الملخص

**المقدمة:**

متلازمة المحتال (IS) هي ظاهرة نفسية تتمثل في الشك المستمر في الذات والخوف من اكتشاف عدم الكفاءة، على الرغم من وجود أدلة واضحة على الكفاءة. هدفت هذه الدراسة إلى تحديد مدى انتشار متلازمة المحتال وتحديد العوامل الديموغرافية المرتبطة بها بين الأطباء المقيمين بتخصص طب الأسرة في مدينة الرياض، المملكة العربية السعودية.

**المنهجية:**

تم اعتماد تصميم الدراسة المقطعية (المستعرضة) ، حيث شملت العينة المستهدفة أطباء الأسرة المقيمين في عدة مراكز تدريبية في الرياض. قام المشاركون باستكمال استبيان ذاتي التعبئة تضمن مقياس كلانس لظاهرة المحتال (CIPS)، بالإضافة إلى جمع بيانات حول الخصائص الاجتماعية والديموغرافية.

**النتائج:**

من بين 268 مشاركًا (بمتوسط عمر 26.1±1.8 عامًا، 63.4% ذكور)، بلغ متوسط درجة متلازمة المحتال 50.8±13.0، بوسيط 51 (IQR: 41–60). أظهرت النتائج أن أكثر من نصف المشاركين (53.7%، ن=144) وقعوا ضمن فئة المتلازمة المتوسطة، بينما أبلغ 23.1% (ن=62) عن خصائص قليلة لمتلازمة المحتال، و21.3% (ن=57) عن تكرارها بشكل ملحوظ، في حين عانى 1.9% (ن=5) من أعراض شديدة. كما لوحظ اختلاف معنوي في توزيع فئات المتلازمة بين مراكز التدريب (p<0.001). وُجدت علاقة معنوية بين الحالة الاجتماعية وشدة متلازمة المحتال (p=0.037)، حيث أظهر المتزوجون خصائص أقل للمتلازمة (30.3%) مقارنة بالعزاب (20.8%). ومع ذلك، لم يكن هناك ارتباط معنوي بين كل من العمر (p=0.684) أو الجنس (p=0.714) وفئات المتلازمة. كما لم يكن لكون الطبيب من الجيل الأول تأثير معنوي على الإصابة بالمتلازمة (p=0.461).

**الاستنتاج:**

أظهرت الدراسة انتشارًا مرتفعًا لمتلازمة المحتال بين الأطباء المقيمين بتخصص طب الأسرة في الرياض، حيث عانى معظمهم من أعراض متوسطة إلى متكررة. قد يساعد التركيز على العوامل مثل الحالة الاجتماعية للأطباء المقيمين وتقديم دعم نفسي قوي في تقليل حدة المتلازمة. توصي الدراسة المستقبلية باستخدام تصاميم طولية لاستكشاف المسارات السببية وتوسيع نطاق البحث ليشمل تخصصات و مناطق طبية أخرى.

**الكلمات المفتاحية:**

طب الأسرة، طبيب مقيم، برنامج الإقامة، متلازمة المحتال.

## Introduction

Imposter syndrome (IS) is a psychological phenomenon characterized by persistent self-doubt and an internalized fear of being exposed as a fraud, despite clear evidence of competence and achievements. Individuals with IS often attribute their success to external factors, such as luck or timing, rather than their own abilities.^1^ This syndrome is widespread across professions, and as many as 70 % of individuals have been estimated to experience IS at some point in their careers.^2^^,^^3^ IS is particularly pronounced in high-pressure environments such as healthcare, in which it can exacerbate stress and decrease professional confidence.^4^, ^5^, ^6^, ^7^ The prevalence of IS has been found to be significantly higher among U.S. physicians than the general U.S. working population, particularly because of the high expectations and systemic pressures in the medical field.^8^^,^^9^ Despite its substantial prevalence, IS remains absent from formal diagnostic frameworks such as the ICD or DSM, thus underscoring the need for greater recognition and study of this condition.^10^

The medical profession is particularly susceptible to IS because of its high-stakes nature, steep learning curves, and continual evaluations.^11^^,^^12^ IS has been found to be prevalent among healthcare professionals, and physicians and medical trainees frequently report feelings of inadequacy and fear of failure despite extensive training and qualifications.^8^^,^^9^^,^^13^^,^^14^ In one study, nearly one-third of family medicine residents experienced IS, and more than 75 % expressed doubts regarding their preparedness to practice, despite feeling adequately trained.^15^ IS has been associated with substantial mental health challenges in medical professionals, including anxiety, depression, and burnout, thus further exacerbating the pressures inherent to the field.^16^ Nonetheless, IS among practicing physicians, particularly in KSA, remains an important understudied gap in the literature.^17^

Global understanding of IS continues to grow, yet research examining IS in KSA is scarce, particularly among family medicine residents. However, gaining knowledge regarding IS is important in KSA, whose healthcare training system faces unique cultural obstacles and institutional barriers.^18^^,^^19^ The aim of this study was to investigate the prevalence of IS among family medicine residents in Riyadh, KSA, and to identify key demographic and professional predictors.

## Materials and Methods

### Study design and setting

This cross-sectional, observational study was conducted among family medicine residents at multiple training centers in Riyadh, KSA. Data collection occurred over 3 months, during which the participants completed a structured, self-administered questionnaire aimed at determining the prevalence of IS and identifying its predictors.

### Eligibility and data collection

The target population included family medicine residents in Riyadh. Specialists and consultants were excluded to ensure uniformity of the participant group, thereby enabling identification of residency-specific stressors and development obstacles within this field. Participants who refused to provide consent to participate or had scores of ≥3 on the Patient Health Questionnaire-2 (PHQ-2), a screening tool for depression, or the Generalized Anxiety Disorder-2 (GAD-2) questionnaire, a screening tool for generalized anxiety disorder, were excluded from the study, to minimize the effects of confounding factors of other underlying mental health conditions on the IS assessment. Data were collected with an online questionnaire deployed through Google Forms.

### Study questionnaire

The survey included three sections. The demographics section gathered data on participant age, gender, training center, marital status, and residency level. The Clance Impostor Phenomenon Scale (CIPS), a 20-item instrument, was used to assess IS severity.^20^ The questionnaire includes 20 Likert scale items, each rated on a scale from 1 (not at all true) to 5 (very true). According to the total score, the IS symptoms were rated by severity (few, ≤40; moderate, 41–60; frequent, 61–80; and intense, >80).

Mental health was evaluated with the PHQ-2 and GAD-2, each consisting of two items rated from 0 (not at all) to 3 (nearly every day), thus resulting in a total score range of 0–6. GAD-2 scores ≥3 suggest potential generalized anxiety disorder, whereas PHQ-2 scores ≥3 indicate possible major depressive disorder.^21^ Higher scores reflect greater symptom severity. Both tools were adapted from validated sources, and permissions were secured as required. The questionnaire was administered in English without modifications. Respondents with PHQ-2 or GAD-2 scores ≥3 were excluded from the analysis.

### Ethical approval and consent

Ethical approval for the study was obtained from the King Fahad Medical City Institutional Review Board. Participation was voluntary, and the first survey page provided an invitation explaining the study's purpose of measuring IS prevalence and its effects on professional performance, mental health, and job satisfaction among primary healthcare physicians. The estimated completion time was 3–5 min. Proceeding with the survey implied consent to the use of responses in research. Confidentiality was maintained, and participants were informed that they could withdraw from the study at any time.

### Sample size calculation

The sample size was estimated according to an assumed prevalence of 20 % for IS, with a 95 % confidence level and a 5 % margin of error. According to Raosoft's sample size calculator, the minimum required sample size was determined to be 246 participants. To account for potential non-response or incomplete data, the survey was distributed to all eligible family medicine residents practicing in Riyadh City during the data collection period.

### Statistical analysis

Data analysis was performed in R v4.3.^22^ Categorical variables are summarized as frequency and percentage, and continuous variables are expressed as mean and standard deviation, or median and interquartile range (IQR) for non-normally distributed data. Associations between categorical variables were assessed with chi-square tests, whereas t-tests were used for continuous, normally distributed variables. The Mann–Whitney test was used to compare non-normally distributed variables between groups. Spearman's correlation was used to assess associations between continuous and ordinal variables. The statistical significance threshold was set at p < 0.05.

## Results

The demographic characteristics of the study participants (N = 268) are summarized in Table 1. Most participants were 24–29 years of age (94.4 %, n = 253), whereas a smaller proportion was 30–34 years of age (5.6 %, n = 15). Gender distribution analysis showed that 63.4 % (n = 170) were men, whereas women constituted 36.6 % (n = 98) of the participants. Most participants had single marital status (75.4 %, n = 202), whereas 24.6 % (n = 66) reported being married. A substantial proportion of the participants were first-generation physicians (70.5 %, n = 189), whereas 29.5 % (n = 79) reported having family members in the medical profession. The participants were distributed across three residency levels, among which R1 was most represented (39.9 %, n = 107), and was followed by R2 (36.2 %, n = 97) and R3 (23.9 %, n = 64).

**Table 1:** Sociodemographic characteristics of family medicine residents in riyadh, KSA.

Characteristic	*Category*	*Frequencies (n) and Percentages*
**Age (years)**	24–29	253 (94.4 %)
	30–34	15 (5.60 %)
**Gender**	Female	98 (36.6 %)
	Male	170 (63.4 %)
**Marital status**	Married	66 (24.6 %)
	Single	202 (75.4 %)
**First-generation physician**	No	79 (29.5 %)
	Yes	189 (70.5 %)
**Residency level**	R1	107 (39.9 %)
	R2	97 (36.2 %)
	R3	64 (23.9 %)
**Training center**	King Abdulaziz medical City–NGHA Riyadh	16 (5.97 %)
	King Abdullah training center hospital–Princess Norah training center	9 (3.36 %)
	King Khalid training center hospital–Riyadh	30 (11.2 %)
	Prince Sultan military medical city–Riyadh	30 (11.2 %)
	Riyadh first health cluster	66 (24.6 %)
	Riyadh Second health cluster	101 (37.7 %)
	Security forces hospital–Riyadh	16 (5.97 %)
**Total Number**		N = 268

The participants’ institutional affiliations were diverse. The largest proportion of participants came from Riyadh Second Health Cluster (37.7 %, n = 101), which was followed by Riyadh First Health Cluster (24.6 %, n = 66). King Khalid Training Center Hospital and Prince Sultan Military Medical City each contributed 11.2 % (n = 30) of the sample, whereas King Abdulaziz Medical City–NGHA Riyadh and Security Forces Hospital–Riyadh each accounted for 5.97 % (n = 16). The least represented institution was King Abdullah Training Center Hospital.

**Figure 1:**
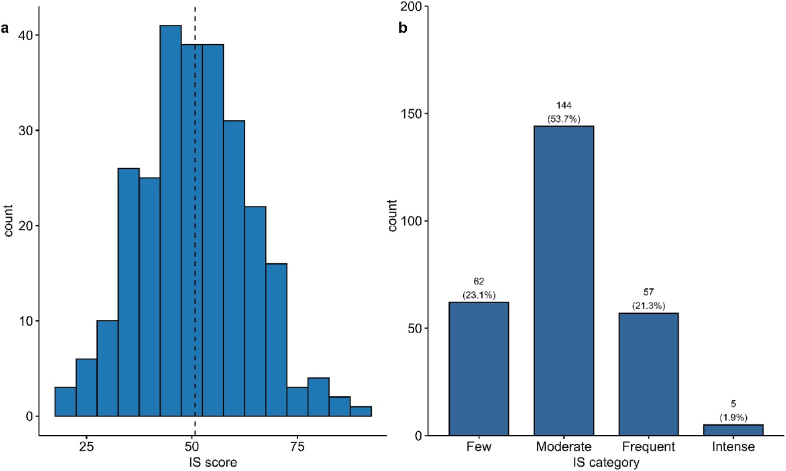
Distribution of Impostor Syndrome (IS) a) Scores and b) Categories. The left panel (a) shows the histogram of IS scores among participants, whereas the right panel (b) presents the distribution of participants across IS symptom severity categories (few, moderate, frequent, and intense).

The median IS (Figure 1a) score was 51 (interquartile range: 41–60), and the mean score was 50.8 ± 13.0. Analysis of IS categories (Figure 1b) indicated that most participants (53.7 %, n = 144) were in the moderate IS category, whereas 23.1 % (n = 62) were in the few IS category (minimal feelings of being an impostor), and 21.3 % (n = 57) were in the frequent IS category (regular feelings of being an impostor). A smaller subset of participants (1.9 %; n = 5) exhibited intense IS (severe and pervasive impostor syndrome experiences). Therefore, although most participants experienced moderate IS tendencies, a smaller but notable portion reported frequent or intense IS symptoms.

**Figure 2:**
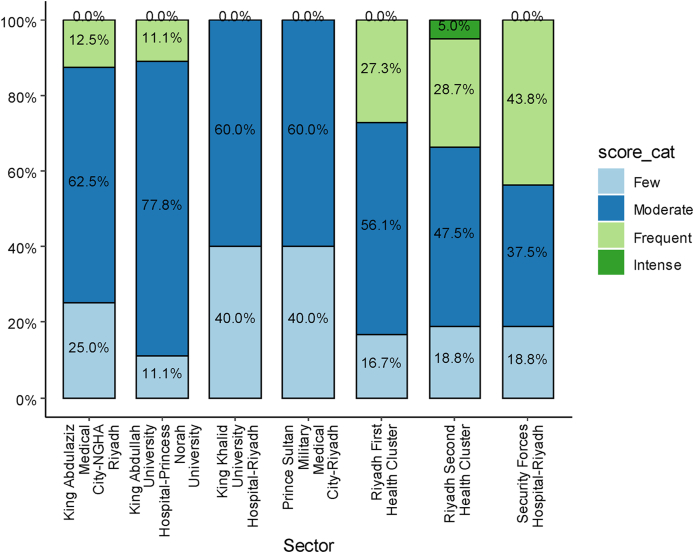
Distribution of Impostor Syndrome Categories by Sector. The bar chart illustrates the percentage distribution of participants across impostor syndrome severity categories (few, moderate, frequent, and intense) for each sector. Colors represent IS categories (few, light blue; moderate, dark blue; frequent, green; intense, dark green). Percentages are displayed within each bar.

The distribution of IS categories varied significantly across sectors (Figure 2, **p < 0.001**). At King Abdulaziz Medical City–NGHA Riyadh, 62.5 % of participants were in the moderate IS category, 25.0 % were in the few IS category, 12.5 % were in the frequent IS category, and no participants were classified as having intense IS. Similarly, at King Abdullah Training Center Hospital–Princess Norah Training Center, 77.8 % of participants were classified in the moderate category, 11.1 % each were classified in the few and frequent categories, and no participants reported intense IS. At King Khalid Training Center Hospital–Riyadh and Prince Sultan Military Medical City–Riyadh, the participants were equally distributed between the moderate (60.0 %) and few (40.0 %) IS categories, and no participants reported frequent or intense IS.

At the Riyadh First Health Cluster, 56.1 % of participants were in the moderate category, 27.3 % were in the frequent category, 16.7 % were in the few category, and no intense IS was reported. At the Riyadh Second Health Cluster, the largest proportion (47.5 %) of participants were in the moderate category, 28.7 % were in the frequent category, and 18.8 % each were in the few and intense categories. Finally, at Security Forces Hospital–Riyadh, 43.8 % of participants were in the frequent category, 37.5 % were in the moderate category, 18.8 % were in the few category, and no intense IS cases were found. These findings highlighted the variation in IS severity levels across sectors: moderate IS was most common across sectors, and frequent IS was markedly higher in specific sectors, as observed at Security Forces Hospital–Riyadh.

The associations between IS categories and demographic variables are shown in Table 2. **Age** showed no significant association with IS categories (**p = 0.684**). Among participants 24–29 years of age, 23.7 % were in the few category, 53.0 % were in the moderate category, and 23.3 % were in the frequent category. Among participants >29 years of age, 13.3 % were in the few category, 66.7 % were in the moderate category, and 20.0 % were in the frequent category. G**ender** was also not significantly associated with IS category (**p = 0.714**). The distribution among women was few, 20.4 %; moderate, 55.1 %; and frequent, 24.5 %. The distribution among men was few, 24.7 %; moderate, 52.9 %; and frequent, 22.4 %.

**Table 2:** Associations between sociodemographic factors and impostor syndrome severity.

Variable	Few	Moderate	Frequent	p-value (overall)
	*N = 62*	*N = 144*	*N = 62*	
**Age (years)**				
24–29	60 (23.7 %)	134 (53.0 %)	59 (23.3 %)	0.684
>29	2 (13.3 %)	10 (66.7 %)	3 (20.0 %)	
**Gender**				
Female	20 (20.4 %)	54 (55.1 %)	24 (24.5 %)	0.714
Male	42 (24.7 %)	90 (52.9 %)	38 (22.4 %)	
**Marital status**				
Married	20 (30.3 %)	36 (54.5 %)	10 (15.2 %)	0.037
Single	42 (20.8 %)	108 (53.5 %)	52 (25.7 %)	
**Residency level**				
R1	18 (16.8 %)	63 (58.9 %)	26 (24.3 %)	0.258
R2	24 (24.7 %)	52 (53.6 %)	21 (21.6 %)	
R3	20 (31.2 %)	29 (45.3 %)	15 (23.4 %)	
**First-generation physician**				
No	16 (20.3 %)	41 (51.9 %)	22 (27.8 %)	0.461
Yes	46 (24.3 %)	103 (54.5 %)	40 (21.2 %)	

∗Analysis was performed with chi-square test of independence or Fisher's exact test.

**Marital status** was significantly associated with IS category (**p = 0.037**). Married participants were more likely to be in the few category (30.3 %) and less likely to be in the frequent category (15.2 %) than single participants (few, 20.8 %; frequent, 25.7 %). **Residency level** was not significantly associated with IS category (**p = 0.258**). Among R1 participants, 16.8 % were in the few category, 58.9 % were in the moderate category, and 24.3 % were in the frequent category. R2 participants showed similar proportions: few, 24.7 %; moderate, 53.6 %; and frequent, 21.6 %. In contrast, a higher proportion of R3 participants were in the few category (31.2 %), whereas 45.3 % were in the moderate category, and 23.4 % were in the frequent category. **First-generation physician status** showed no significant association with IS category (**p = 0.461**). Participants who were not first-generation physicians were distributed as follows: few, 20.3 %; moderate, 51.9 %; and frequent, 27.8 %. Participants who were first-generation physicians were distributed as follows: few, 24.3 %; moderate, 54.5 %; and frequent, 21.2 %.

## Discussion

### Prevalence and effects of IS

IS not only influences individual well-being but also has extensive implications in professional performance and patient care. Physicians experiencing IS might have low self-confidence, thus hindering decision-making, professional growth, and leadership potential.^23^ Herein, IS was found to be highly prevalent among family medicine residents in Riyadh: more than half of the participants experienced moderate IS, and one-quarter experienced frequent IS. These findings align with global trends highlighting the widespread nature of IS among medical trainees. In one study, 62.8 % of medical students and 57.2 % of residents in China experienced significant IS, which is often associated with burnout and mental health challenges.^14^ In another study in Pakistan, 88.5 % of surgical residents exhibited moderate-to-intense IS, and female residents had a higher prevalence than males.^24^ Alzufari and colleagues have observed a similar trend at the University of Sharjah: 46.4 % of medical students experienced moderate IS, 35.8 % experienced frequent IS; moreover, IS was often exacerbated by academic pressure and peer pressure.^25^

### Institutional factors and professional predictors

IS is a major challenge in medical education and training environments, where steep learning curves and high expectations might contribute to its prevalence.^26^, ^27^, ^28^ Moreover, IS has been associated with heightened risks of anxiety, depression, and even suicidal ideation, particularly in high-pressure medical environments.^29^ These mental health challenges can decrease productivity and increase the likelihood of burnout, thus further straining healthcare systems.^9^

Our study also revealed significant variability in IS severity across training centers; consequently, institution-specific factors, such as workload intensity, leadership styles, or support systems, might have contributed to our findings. Several studies have corroborated the major role of institutional factors in shaping IS experiences. **Amir** and colleagues have found that mentorship, workload, and the availability of extracurricular activities significantly influence IS prevalence among postgraduate trainees, and have emphasized the importance of supportive institutional environments.^30^ In another study, competitive peer environments and advisor attitudes exacerbated IS among medical students at the University of Sharjah, and 35.8 % of students experienced frequent IS.^25^

In Thailand, transitions between training stages and poor mental health support have been found to significantly contribute to IS among clinical students, thereby highlighting the importance of stress management in decreasing IS severity.^12^ Furthermore, **Teplinsky has** reported that gaps in professional mentorship and systemic challenges within institutional cultures significantly contribute to IS among oncology trainees, and IS is exacerbated by additional stressors such as social media use.^31^ **Lin and colleagues have also** identified that IS among surgeons is associated with institutional intolerance of uncertainty and lack of confidence-building support, thus underscoring a need for targeted interventions.^32^ Together, previous findings suggest that addressing institutional factors, such as by fostering mentorship programs, promoting a culture of positive feedback, and improving work-life balance, have the potential to significantly mitigate IS prevalence. Tailored, institution-specific strategies are essential to enhance the well-being of medical trainees and limit the effects of IS on professional development.

### Demographic predictors

This study identified significant associations between IS severity and certain demographic and professional predictors, including marital status. Married participants were more likely to report few IS symptoms (30.3 %) than single participants (20.8 %). IS severity was significantly associated with marital status: married residents experienced lower IS levels than single residents. Spousal emotional and social connections appear to help residents resist self-doubt and stress, which are common medical training experiences. The married residents might have demonstrated lower IS severity because marital relationships provided stability and helped them navigate professional challenges. Similar findings have been reported in **other studies, in which** married medical students exhibited lower IS prevalence than single medical students, because of greater emotional stability and familial support.^27^^,^^33^^,^^34^ Residency level was another critical factor: first-year residents exhibited the highest proportion of moderate IS (58.9 %). Similar patterns have been observed in another study, in which transitions into clinical training were identified as significant stressors contributing to elevated IS prevalence in early training stages.^35^ Another study has indicated that career transitions and professional challenges further exacerbate IS severity during early residency.^36^

Herein, first-generation physician status was not significantly associated with IS severity. However, **Araújo and colleagues** have identified that critical family dynamics and external pressures are significant contributors to IS development among medical students.^37^ Similarly, **El-Setouhy and colleagues** have demonstrated that family income and parental education are associated with IS prevalence, such that less supportive home environments are associated with higher IS scores among medical trainees.^38^, ^39^, ^40^

### Implications and recommendations

Our findings suggested that addressing predictors such as marital status, residency year, and familial influences through tailored interventions might significantly decrease IS prevalence. Support strategies, particularly during transitional training stages, will be essential to foster resilience and professional confidence in medical trainees.

## Limitations

This study provides valuable insights into the prevalence and predictors of IS among family medicine residents in Riyadh. However, several limitations should be acknowledged. First, the cross-sectional design restricted the ability to infer causation between identified predictors and IS. However, other research designs across time allows groups to monitor changes, thus enhancing understanding of causal elements. Second, the use of self-reported questionnaires might have introduced response bias, because participants might have underreported or exaggerated their experiences. Third, the study focused on family medicine residents in Riyadh, thereby limiting the generalizability of findings to other specialties or regions. Future research should consider longitudinal designs, larger sample sizes, and more diverse qualitative methods to explore IS in greater depth.

## Conclusion

A high prevalence of IS was observed among family medicine residents in Riyadh, who had moderately to frequently occurring symptoms. Social support appeared to be a factor in IS: because the levels of IS differed according to marital status, relationships might protect against IS. These results highlight the importance of analyzing how institutional and psychosocial factors might influence IS prevalence among residents. The current study might provide a basis for future research using longitudinal and interventional methods to advance knowledge of such relationships, as well as to assess the effects of interventions such as mentorships and mental health programs on lessening the effects of IS.

## Authors contributions

Dr. Omar Alghamdi: supervision, critical revision of the manuscript, and final approval; Dr. Salem Alshahrani: data collection, introduction writing, and literature review; Dr. Mohammed Alqahtani: data collection, introduction writing, and discussion writing; Dr. Abdulelah Alotaibi: data collection, study conceptualization, and conclusion writing; Dr. Abdulrahman Bamhair: data collection, methods, abstract writing, formatting of the manuscript, and management; All authors supported the creation of the final manuscript and accept full responsibility for the entire study content. All authors have critically reviewed and approved the final draft and are responsible for the content and similarity index of the manuscript.

## Funding

No funding or grant were received for conducting this study.

## Conflict of interest

The authors have no conflicts of interest to declare.

## Appendix

IS questions

• **IS01**: I have often succeeded on a test or task even though I was afraid that I would not do well before I undertook the task.
• **IS02**: I can give the impression that I'm more competent than I really am.
• **IS03**: I avoid evaluations if possible and have a dread of others evaluating me.
• **IS04**: When people praise me for something I've accomplished, I'm afraid I won't be able to live up to their expectations of me in the future.
• **IS05**: I sometimes think I obtained my present position or gained my present success because I happened to be in the right place at the right time or knew the right people.
• **IS06**: I'm afraid people important to me may find out that I'm not as capable as they think I am.
• **IS07**: I tend to remember the incidents in which I have not done my best more than those times I have done my best.
• **IS08**: I rarely do a project or task as well as I'd like to do it.
• **IS09**: Sometimes I feel or believe that my success in my life or in my job has been the result of some kind of error.
• **IS10**: It's hard for me to accept compliments or praise about my intelligence or accomplishments.
• **IS11**: At times, I feel my success has been due to some kind of luck.
• **IS12**: I'm disappointed at times in my present accomplishments and think I should have accomplished much more.
• **IS13**: Sometimes I'm afraid others will discover how much knowledge or ability I really lack.
• **IS14**: I'm often afraid that I may fail at a new assignment or undertaking even though I generally do well at what I attempt.
• **IS15**: When I've succeeded at something and received recognition for my accomplishments, I have doubts that I can keep repeating that success.
• **IS16**: If I receive a great deal of praise and recognition for something I've accomplished, I tend to discount the importance of what I've done.
• **IS17**: I often compare my ability to those around me and think they may be more intelligent than I am.
• **IS18**: I often worry about not succeeding with a project or examination, even though others around me have considerable confidence that I will do well.
• **IS19**: If I'm going to receive a promotion or gain recognition of some kind, I hesitate to tell others until it is an accomplished fact.
• **IS20**: I feel bad and discouraged if I'm not “the best” or at least “very special” in situations that involve achievement.
